# Acceptability of 12 fortified balanced energy protein supplements ‐ Insights from Burkina Faso

**DOI:** 10.1111/mcn.13067

**Published:** 2020-08-05

**Authors:** Leslie Jones, Brenda de Kok, Katie Moore, Saskia de Pee, Juliet Bedford, Katrien Vanslambrouck, Laeticia Celine Toe, Carl Lachat, Nathalie De Cock, Moctar Ouédraogo, Rasmané Ganaba, Patrick Kolsteren, Sheila Isanaka

**Affiliations:** ^1^ Anthrologica Oxford UK; ^2^ Department of Food Technology, Safety and Health, Faculty of Bioscience Engineering Ghent University Ghent Belgium; ^3^ Applying Evidence for Nutrition (AE4N) Wassenaar The Netherlands; ^4^ Nutrition Division World Food Programme Rome Italy; ^5^ Division of Food and Nutrition Policy and Programs Friedman School of Nutrition Science and Policy, Tufts University Boston Massachusetts USA; ^6^ Institut de Recherche en Sciences de la Santé (IRSS), Unité de nutrition et maladies métaboliques Ghent University Ghent Belgium; ^7^ AFRICSanté Bobo‐Dioulasso Burkina Faso; ^8^ Department of Nutrition Harvard T.H. Chan School of Public Health Boston Massachusetts USA

## Abstract

Poor maternal nutrition contributes to poor birth outcomes, including low birth weight and small for gestational age births. Fortified balanced energy protein (BEP) supplements may be beneficial, although evidence is limited. This mixed method study, conducted among pregnant women in Burkina Faso, is part of a larger clinical trial that seeks to understand the impact of fortified BEP supplements on pregnancy outcomes and child growth. The formative research reported here, a single‐meal rapid assessment of 12 product formulations, sought to understand product preferences for provision of BEP supplements and contextual factors that might affect product acceptability and use. Results indicate a preference for products perceived as sweet rather than salty/savoury and for products perceived as familiar, as well as a sensitivity to product odours. Women expressed a willingness and intention to use the products even if they did not like them, because of the health benefits for their babies. Data also indicate that household food sharing practices may impact supplement use, although most women denied any intention to share the products. Sharing behaviour should therefore be monitored, and strategies to avoid sharing should be developed during the succeeding parts of the research.

**Key messages**

- Current evidence indicates that balanced energy protein supplements may be effective in improving birth outcomes, but more needs to be known about preferred types of supplements and potential facilitators and barriers to product use.
- Although women in this study preferred products with familiar flavours that are perceived as sweet rather than savoury/salty, they expressed a willingness to eat any product they can tolerate because of the potential health benefits for their baby.
- Expectations regarding sharing of foods may impact supplement use; although women expressed an intention to avoid sharing of the supplements, sharing behaviours should be carefully monitored and strategies developed to help women avoid sharing.

## 1 INTRODUCTION

Low birth weight (LBW) is a significant risk factor for infant mortality, estimated to account directly or indirectly for 60%–80% of neonatal deaths worldwide (Katz et al., 2014). Poor maternal nutrition status, including energy and micronutrient deficiencies, contributes to poor birth outcomes (da Silva Lopes et al., 2017; Fall, Fisher, Osmond, & Margetts, 2009; Gernand, Schulze, Stewart, West, & Christian, 2016). Women who enter pregnancy with low body mass index (BMI) or short stature are at increased risk of adverse health outcomes (Rahman et al., 2015) as well as small for gestational age (SGA) births (Kozuki et al., 2015).

Although Burkina Faso has seen significant improvements in maternal and infant health over the last 25 years, the infant mortality rate has remained high, at 53 per 1000 live births in 2016 (Hug, Sharrow, & You, 2017). Of those babies with a reported birth weight (63.6% of all births), 13.9% were LBW (INSD & ICF Intl., 2012); a previous study by members of this research team estimated that the number of SGA births is between 32.2% and 41.6% in the Houndé district (Huybregts et al., 2009; Roberfroid et al., 2008). The overall prevalence of underweight in women of reproductive age in Burkina Faso is 15.7%, although prevalence is nearly twice that (31.1%) in some regions (INSD & ICF Intl., 2012).

Evidence indicates that providing pregnant women with a balanced energy–protein (BEP) food supplement may have a positive effect on birth outcomes (Imdad & Bhutta, 2011 ; Ota, Hori, Mori, Tobe‐Gai, & Farrar, 2015), particularly among undernourished women. World Health Organisation antenatal care guidelines recommend provision of BEP supplements in populations where the prevalence of undernourished women (low BMI) is greater than 20% (WHO, 2016). In addition, recent reviews support the conclusion that multiple micronutrient supplements (MMS) are beneficial in countries with a high prevalence of multiple micronutrient deficiencies (Bourassa et al., 2019; Keats, Haider, Tam, & Bhutta, 2019; Smith et al., 2017). Transitioning from iron–folic acid supplements to MMS has also been found to be cost‐effective (Kashi et al., 2019; Nutrition International, 2019).

Guidance from the Bill & Melinda Gates Foundation (BMGF) therefore recommends development of a multimicronutrient fortified BEP supplement as a ready‐to‐consume product (BMGF, 2016). However, more research is needed to quantify the impact of specific fortified BEP supplements on birth outcomes, as well as to understand how factors such as product preferences and community influences on product use may affect acceptability and uptake of specific supplements in a given context.

This paper focuses on the first part of phase 1 of the ‘Micronutriments et Santé de la Mère et de l'Enfant III’ (MISAME III) study, a two‐phase study seeking to evaluate the preferred product type for fortified BEP supplements (phase 1) and their impact on pregnancy outcomes and child growth in Burkina Faso (phase 2) (clinicaltrials.gov identifier NCT03533712). Data collection activities during this part of phase 1 sought to (1) assess the hedonic properties of 12 formulations of fortified BEP supplements, (2) identify preferred product type(s) for fortified BEP supplements (for further testing in the second part of phase 1) and (3) assess the acceptability, general preferences, advantages and barriers across product types.

## 2 METHODS

### 2.1 Study design

Data were collected using a convergent mixed methods approach that included quantitative and qualitative tools (Creswell & Plano Clark, 2011). The value of gathering mixed methods data is the mutual validation of results through a process of triangulation. Throughout this research, the convergence of the different methods allowed for testing the same hypothesis and answering the same part of a research question through multiple lenses (Lobe, 2008).

### 2.2 Recruitment and data collection

Data were collected over 3 weeks between May and June 2018 in Houndé district, Burkina Faso, West Africa. The district was selected because of the high percentage of SGA births and the study team's prior experience in the area, which facilitated study logistics. Houndé has one hospital and 31 health centres, of which five were identified for inclusion in this study because of their accessibility even during the rainy season and willingness to participate. All pregnant women aged 15 to 35 who visited one of the five health facilities for prenatal consultation were invited by their health care providers to participate. The only exclusion criteria were allergies to product ingredients (soy, dairy products, eggs, gluten and nuts), none of which were reported during recruitment. Eight women per health centre (40 in total) were targeted for inclusion in the study, and recruitment ceased when this number were enrolled. On the first day of data collection, which took place at each of the five health centres, data collectors explained the study and obtained informed consent from all participants. Women were provided with lunch in consideration of their participation.

### 2.3 Ethical statement

The study was approved by the ethics committees of Centre Muraz, Burkina Faso, the Harvard T.H. Chan School of Public Health and the Ghent University Hospital, Belgium. The study was explained in detail, and participants willing to take part completed the consent form with a signature or thumbprint. Informed assent was obtained from participants under the age of 18, and consent of those participants' husband or guardian was also acquired.

### 2.4 Tested supplements

The nutritional composition of the specific BEP supplements was established during an expert consultation convened at the BMGF in September 2016 (Bill & Melinda Gates Foundation, 2016) and is set forth in Table S1. The research partners liaised with private sector partners to develop supplements in seven different product formats to be evaluated in formative research: biscuits, bars, filled sticks, cold beverages, a soup, lipid‐based pastes and ‘pillows’, a crispy puffed snack formulation. The pastes, bars, biscuits and pillows were available in both a primarily sweet and a primarily savoury flavour profile. Several of the flavours were developed specifically to evoke familiar flavours from the Burkinabè diet, such as the fermented drink, the mango bars and the tomato/onion bars. Table 1 presents the 12 products tested in the study and the flavour profiles and manufacturers of each.

**TABLE 1:** Product groupings and manufacturers

Product name	Product grouping	Product manufacturer
Sweet lipid‐based paste	Sweet	Nutriset
Mango bar	Sweet	Nutriset
Vanilla‐filled sticks	Sweet	Nutriset
Vanilla biscuits	Sweet	Nutriset
Vanilla drink	Sweet	Nutriset
Unseasoned pillows^a^	Sweet	Mars
Fermented drink^a^	Sweet	DSM
Tomato and onion lipid‐based paste	Savoury	Nutriset
Tomato and onion bar	Savoury	Nutriset
Tomato and onion biscuits	Savoury	Nutriset
Chicken soup	Savoury	Nutriset
Seasoned pillows	Savoury	Mars

^a^ The unseasoned pillows and the fermented drink did not have a sweet taste but were grouped with the sweet products to distinguish them from products containing savoury flavours.

### 2.5 Research tools

The quantitative (hedonic testing and product ranking exercises) and qualitative (focus group discussions) tools were pretested with a subset of volunteers (pregnant women) from the local community and were refined over the course of several weeks.

#### 2.5.1 Quantitative tools

The quantitative data were collected electronically using the Census and Survey Processing System (CSPro) data management programme (Version 7.1; United States Census Bureau, 2017). Over two consecutive days, women were presented with samples (in the amount of 25% of the full daily portion) of each of the 12 products. Products were divided into sweet and savoury groups, with seven characterised as sweet and five as savoury. The products were moderately sweetened; as presented in Table S2, added sugars ranged from 13.7 to 21.6 g per 100‐g serving for the sweet products and from 0 to 13 g per 100 g for the savoury products. This level contributes 2.5%–3.9% of energy from sugar in a 2200‐kcal diet for the sweet products and 0%–2.4% for the savoury products. However, certain products were grouped as ‘sweet’ in order to distinguish them from those with a more savoury taste profile.

Acceptability of five savoury products was assessed on day one of data collection and of seven sweet products on day two. The amount of each product consumed (by weight) and the time taken were recorded; a limit of 20 min was allowed for the consumption of each individual product. Each woman was asked a series of questions about the acceptability and hedonic characteristics of each of the 12 products in turn after consumption, using a 7‐point Likert scale to answer from 1 (*I dislike it very much*) to 7 (*I like it very much*) (Krosnick & Presser, 2010). The women were also presented with a series of statements regarding their potential use of the product and willingness to consume it during pregnancy, and the responses were scaled from 1 (*I do not agree at all*) to 7 (*I agree completely*). The 7‐point scale was graphically depicted using a range of emoticon faces (very unhappy to very happy).

Following individual evaluation of each product grouping (i.e. sweet or savoury), participants were asked to rank that group's products in order of preference from ‘most liked’ to ‘least liked’ for each of taste, texture, smell, colour, portion size (full serving), ease of use and overall preference. Participants were also asked individually on day two to identify their overall ‘top three’ products out of all 12 products tasted. All quantitative data were collected from participants in individual sessions so that they were unable to hear (and potentially be influenced by) others' responses to product acceptability, use and individual product ranking questions.

#### 2.5.2 Qualitative tools

Complementary qualitative data were also collected on a third consecutive day of data collection in a series of 5 eight‐person focus group discussions, composed of the women who had participated in the previous days' product testing. Women were grouped together roughly by age (younger women together and older women together) to reduce potential age‐related impediments to free expression (Hughes & Dumont, 1993). A structured framework was used to elicit contextual data relevant to women's general perspectives on product use and dietary practices. The framework included questions related to factors that might influence acceptability and consumption of flavour profiles, as well as sharing dynamics, local food practices and potential supplement use. The focus groups, which were audio recorded, were conducted with one moderator (a trained sociologist) and one note taker who supported with additional observational notes that would not be captured through the audio recording.

An additional ranking exercise was included in the focus group discussion to elicit further narratives around characteristics affecting the potential use of the products and how those characteristics related to each other. Participants were then asked to discuss and reach consensus on their top three products as a group.

### 2.6 Data analysis

The 7‐point Likert scale used for quantification of product acceptability and perceptions was treated as a continuous variable (Sullivan & Artino, 2013). The mean (± SD) was calculated for the hedonic characteristics of acceptability, perception of product use and willingness to use for 12 months. Amount of money willing to pay and perception of portion size were recorded as categorical variables and displayed in numbers and relative percentages. CSPro data files were exported to Stata (Version 14.2; StataCorp, 2015) for statistical analysis.

In order to analyse the product ranking data, a product was awarded three points every time it was ranked first, two points every time it was ranked second and one point every time it was ranked third. If a product was not included in the top three, it received zero points. The points for each product were summed. For the individual ‘top three’ rankings, the maximum possible score was therefore 120 points (40 participants × 3 points maximum) and the minimum was zero (for a product that was never ranked in the ‘top three’). For the focus group rankings, the maximum possible score was 15 points (five focus groups × 3 points maximum) and the minimum was again zero.

Qualitative data were analysed using thematic analysis. This approach allows for the systematic identification and analysis of patterns and themes within a dataset (Braun & Clarke, 2006). Dominant, recurring themes were identified through the review of transcripts and field notes, and a thematic framework was iteratively developed. Salient concepts were then coded by hand and/or using Dedoose (Version 8.2.32; SocioCultural Research Consultants, 2016) and cross‐referenced by the research team for quality assurance. The emerging trends were critically analysed to ensure the emerging themes were relevant to the research objectives (Daly, Kellehear, & Gliksman, 1997): to assess which product types and varieties were preferred and why, what factors affected women's choice of preferred products, how those products would be incorporated into the local diet, the acceptability of snacking and sharing and the acceptability of at‐home consumption of products. The quantitative and qualitative results were then compared and integrated with the final analysis.

## 3 RESULTS

Key demographic data for the 40 study participants are presented in Table 2 below. The mean age was 25.4, with a mean gestational age of 5.2 months. Nearly all (95%, *n* = 38) were married, and 26 (65%) had never attended school.

**TABLE 2:** Demographic characteristics of study participants

Characteristics of pregnant women (*n* = 40)	
Age (mean ± SD)	25.4 ± 4.7
Matrimonial status, *n* (%)	
Married	38 (95%)
Not married	2 (5%)
School attendance, *n* (%)	
None	26 (65%)
Primary	11 (27.5%)
Secondary	3 (7.5%)
Higher education	0 (0%)
Household size, number of people (mean ± SD)	8.7 ± 5.0
Religion, *n* (%)	
Christian	21 (52.5%)
Muslim	14 (35%)
Animist	5 (12.5%)
Gestational age in months (mean ± SD)	5.2 ± 1.9
First pregnancy, *n* (%)	7 (17.5%)
Number of children (mean ± SD)	1.9 ± 1.4
Number of pregnancy consultations (mean ± SD)	1.8 ± 1.3

### 3.1 Measures of overall preference

Detailed results of the product acceptability and the ranking of individual product characteristics are presented in Table 3 (sweet product grouping) and Table 4 (savoury product grouping). Table 5 presents (1) the results of the individual product ranking activity, (2) the results of the group product ranking activity and (3) the mean individual product acceptability score for the top five products along any of those three metrics.

**TABLE 3:** Hedonic testing, acceptability of sweet products, mean (standard deviation), *n* (%)

	Sweet lipid‐based paste	Vanilla biscuits	Filled sticks	Vanilla drink	Fermented drink	Sweet bar	Unseasoned pillows
	Product consumption, mean (standard deviation)						
Net weight consumed (g)	24.6 (0.9)	17.7 (0.8)^b^	24.6 (0.7)	67.0 (4.9)	63.5 (14.6)	15.4 (3.6)	15.2 (4.2)
Proportion of test portion consumed (%)^a^	98.4	98.3	98.4	95.7	90.7	96.3	89.4
Duration of consumption (min)	3.6 (1.6)	4.0 (1.4)	4.5 (1.4)	3.0 (3.0)	3.8 (4.1)	5.6 (4.5)	7.7 (5.5)
	Appreciation of product (1 = *dislike very much* to 7 = *like very much*), mean (standard deviation)						
Colour	6.7 (0.5)	6.5 (0.7)	6.5 (0.6)	6.3 (1.2)	6.3 (1.1)	6.0 (1.3)	6.2 (1.2)
Taste	6.5 (0.9)	6.6 (0.6)	6.6 (0.5)	6.3 (1.1)	5.9 (1.5)	6.2 (1.0)	5.8 (1.6)
Texture/consistency	6.4 (0.8)	6.4 (0.7)	6.2 (1.0)	6.2 (1.2)	5.9 (1.6)	5.8 (1.2)	5.8 (1.5)
Smell	6.2 (1.0)	6.3 (0.9)	6.2 (0.9)	6.0 (1.4)	6.2 (1.2)	5.5 (1.6)	5.5 (1.6)
Overall appreciation	6.5 (0.7)	6.4 (0.7)	6.4 (0.7)	6.1 (1.1)	6.0 (1.4)	5.8 (1.1)	5.7 (1.4)
Perceived child likeability	6.6 (0.6)	6.7 (0.5)	6.5 (0.6)	6.3 (0.9)	6.2 (1.2)	6.1 (1.0)	5.7 (1.2)
Perceived adult likeability	6.3 (0.7)	6.3 (0.8)	6.1 (0.8)	6.1 (0.9)	6.0 (1.2)	5.9 (1.0)	5.8 (1.1)
	Perception of product use (1 = *not at all in agreement* to 7 = *very in agreement*), mean (standard deviation)						
Product is convenient to eat	6.3 (0.9)	6.5 (0.7)	6.1 (1.0)	6.2 (1.1)	5.9 (1.5)	5.8 (1.5)	5.7 (1.6)
Product is convenient to eat between meals	6.4 (0.7)	6.6 (0.5)	6.3 (1.0)	6.2 (1.3)	6.0 (1.5)	6.2 (1.1)	5.8 (1.5)
Product is medicine	5.4 (1.8)	5.5 (1.8)	5.5 (1.7)	5.4 (1.8)	5.4 (1.8)	5.5 (1.8)	5.3 (1.7)
Feel full after full portion	5.0 (1.8)	5.1 (2.1)	5.3 (1.7)	4.9 (1.9)	4.7 (2.1)	5.3 (1.7)	5.1 (1.8)
Would share with others	3.4 (2.3)	3.4 (2.2)	3.5 (2.3)	3.4 (2.2)	3.5 (2.2)	3.4 (2.1)	3.6 (2.3)
	Willingness to use daily for 12 months (1 = *not at all in agreement* to 7 = *very in agreement*), mean (standard deviation)						
Would use daily if provided	6.3 (1.0)	6.4 (0.8)	6.2 (1.2)	6.0 (1.5)	6.0 (1.6)	5.6 (1.5)	5.7 (1.7)
Would use daily if purchased	5.8 (1.4)	5.8 (1.3)	5.6 (1.5)	5.5 (1.8)	5.5 (1.9)	5.2 (1.8)	5.1 (2.0)
	Amount willing to pay, *n* (%)						
Would pay how much (CFA)							
0	0 (0%)	0 (0%)	0 (0%)	0 (0%)	0 (0%)	0 (0%)	0 (0%)
1–100	23 (57.5%)	21 (52.5%)	21 (52.5%)	21 (52.5%)	22 (55%)	23 (57.5%)	24 (60%)
101–200	11 (27.5%)	12 (30%)	12 (30%)	11 (27.5%)	11 (27.5%)	11 (27.5%)	8 (20%)
201–300	4 (10%)	3 (7.5%)	4 (10%)	2 (5%)	1 (2.5%)	2 (5.0%)	1 (2.5%)
301–400	1 (2.5%)	0 (0%)	0 (0%)	0 (0%)	0 (0%)	1 (2.5%)	1 (2.5%)
401–500	0 (0%)	2 (5%)	1 (2.5%)	5 (12.5%)	3 (7.5%)	2 (5.0%)	5 (12.5%)
>500	1 (2.5%)	2 (5%)	2 (5%)	1 (2.5%)	3 (7.5%)	1 (2.5%)	1 (2.5%)
	Acceptability of portion size (for a snack), *n* (%)						
Portion size is acceptable	39 (97.5%)	34 (85%)	37 (92.5%)	38 (95%)	34 (85%)	34 (85%)	37 (92.5%)
Too small	1 (2.5%)	5 (12.5%)	1 (2.5%)	2 (5%)	5 (12.5%)	3 (7.5%)	0 (0%)
Too big	0 (0%)	1 (2.5%)	2 (5%)	0 (0%)	1 (2.5%)	3 (7.5%)	3 (7.5%)

^a^ Net weight consumed/sample weight × 100.

^b^ *n* = 38 for weight/duration of consumption for the vanilla biscuits.

**TABLE 4:** Hedonic testing, acceptability of savoury products, mean (standard deviation), *n* (%)

	Savoury lipid‐based paste	Chicken soup	Bar	Biscuits	Seasoned pillows
	Product consumption, mean (standard deviation)				
Net weight consumed (g)	22.9 (5.7)	56.8 (22.6)	14.1 (4.7)	13.1 (5.6)	13.3 (6.0)
Proportion of test portion consumed (%)^a^	91.6	81.1	88.1	77.1	78.2
Duration of consumption (min)	5.8 (5.2)	6.8 (6.9)	8.3 (6.6)	9.4 (6.9)	10.5 (6.7)
	Appreciation of product (1 = *dislike very much* to 7 = *like very much*), mean (standard deviation)				
Colour	6.4 (1.0)	6.0 (1.5)	6.0 (1.2)	6.2 (1.4)	6.0 (1.6)
Taste	6.1 (1.4)	5.7 (1.7)	5.5 (1.6)	5.2 (1.7)	4.8 (2.1)
Texture/consistency	6.0 (1.3)	5.5 (1.8)	5.3 (1.5)	5.2 (1.8)	5.0 (2.1)
Smell	5.7 (1.8)	5.1 (2.2)	5.0 (2.0)	4.9 (2.1)	4.5 (2.4)
Overall appreciation	5.9 (1.5)	5.5 (1.8)	5.3 (1.8)	5.0 (2.1)	5.0 (2.2)
Perceived child likeability	6.2 (1.1)	5.8 (1.4)	6.1 (1.0)	5.9 (1.3)	5.7 (1.7)
Perceived adult likeability	6.0 (1.1)	5.8 (1.2)	5.8 (1.0)	5.5 (1.6)	5.3 (1.8)
	Perception of product use (1 = *not at all in agreement* to 7 = *very in agreement*), mean (standard deviation)				
Product is convenient to eat	6.1 (1.1)	5.8 (1.4)	5.6 (1.5)	5.7 (1.6)	5.5 (1.9)
Product is convenient to eat between meals	6.3 (1.2)	6.0 (1.5)	6.0 (1.4)	5.9 (1.6)	5.4 (1.8)
Product is medicine	5.5 (1.7)	5.5 (1.8)	5.2 (1.8)	5.4 (1.7)	5.1 (1.8)
Feel full after full portion	5.2 (1.6)	5.1 (1.7)	5.2 (1.6)	5.0 (2.0)	5.5 (1.7)
Would share with others	3.4 (2.2)	3.4 (2.2)	3.2 (2.2)	3.7 (2.3)	3.7 (2.4)
	Willingness to use daily for 12 months (1 = *not at all in agreement* to 7 = *very in agreement*), mean (standard deviation)				
Would use daily if provided	5.9 (1.4)	5.5 (1.9)	5.7 (1.6)	5.5 (1.9)	4.9 (2.2)
Would use daily if purchased	5.5 (1.6)	5.1 (2.1)	5.1 (2.0)	5.0 (1.8)	4.5 (2.3)
	Amount willing to pay, *n* (%)				
Would pay how much (CFA)					
0	0 (0%)	0 (0%)	1 (2.5%)	2 (5%)	1 (2.5%)
1–100	18 (45%)	15 (37.5%)	22 (55%)	17 (42.5%)	14 (35%)
101–200	11 (27.5%)	15 (37.5%)	7 (17.5%)	13 (32.5%)	14 (35%)
201–300	4 (10%)	3 (7.5%)	2 (5%)	5 (12.5%)	6 (15%)
301–400	0 (0%)	0 (0%)	1 (2.5%)	0 (0%)	0 (0%)
401–500	4 (10%)	3 (7.5%)	4 (10%)	2 (5%)	2 (5%)
>500	3 (7.5%)	4 (10%)	3 (7.5%)	1 (2.5%)	3 (7.5%)
	Size of portion (for a snack or portion), *n* (%)				
Portion size is acceptable	35 (87.5%)	32 (80%)	33 (82.5%)	29 (74.3%)	27 (67.5%)
Too small	4 (10%)	4 (10%)	2 (5%)	3 (7.7%)	3 (7.5%)
Too big	1 (2.5%)	4 (10%)	5 (12.5%)	7 (18%)	10 (25%)

^a^ Net weight consumed/sample weight × 100.

**TABLE 5:** Top 5 products across three primary metrics

	Vanilla biscuits	Sweet lipid‐based paste	Fermented drink	Vanilla drink	Filled sticks	Unseasoned pillows
Individual product ranking (points)	1 (55)	2 (50)	3 (39)	4 (33)	5 (26)	9 (5)
Group ranking (points)	2 (6)	1 (12)	4 (5)	N/A	2 (6)	5 (1)
Product acceptability (mean score on 7‐point scale)	2 (6.4)	1 (6.5)	5 (6.0)	4 (6.1)	2 (6.4)	7 (5.7)

#### 3.1.1 Sweet product preferences

The quantitative results suggested that participants strongly favoured products they perceived as sweet. The sweet lipid‐based paste and the vanilla biscuit were the top two products according to all three measurements, and the fermented drink, vanilla drink and filled sticks (all sweet products) were consistently in either third, fourth or fifth place. No savoury product was ranked in the top five for any of these three measures.

Qualitative data gathered during the focus group discussions corroborated the quantitative findings regarding participants' preference for sweet products, and this preference was most apparent during direct comparisons between sweet and savoury versions of the same products. When asked specifically to compare the sweet versus the savoury bar, biscuit and lipid‐based paste, the women were virtually unanimous in expressing a preference for the sweet versions of each product. During focus group discussions, women frequently commented on a product's sweet taste as one of its most favourable aspects, and they often said they disliked salty tastes and related products.

Data from focus group discussions also confirmed the women's specific preference for the sweet lipid‐based paste and the vanilla biscuit. Women commented favourably on the paste's sweet taste (‘When you put it in your mouth, its sweet and there is a good smell’) and its ‘milky’ taste (‘I like it, it's as though they've put milk inside’). In contrast to many of the other products, a large number of participants spoke positively about the smell of the lipid‐based paste, whereas others commented favourably on its colour or texture. The majority of participants said that the product was very good as currently formulated and had no changes to suggest. As one woman concluded, ‘When I eat it, I like the smell, the taste, I like everything in this product.’ Comments regarding the vanilla biscuit were similar, although a small minority of women reported disliking the smell: One suggested ‘It's good when we eat it, but it contains an odour that I do not like’ and another confirmed ‘It's as if we have put in garlic.’ Nonetheless, the overall response to the vanilla biscuits was positive, as many women noted, ‘I like everything about it.’

#### 3.1.2 Product odour

Odour was a particularly relevant factor for product preference and impacted several women's ability to tolerate a product. Several participants said that the smell and/or taste of certain products made them nauseous. For every product except one (the fermented drink), odour was the lowest mean Likert score for a hedonic characteristic. Odour was also the only characteristic for which some products received scores of less than 5.0 out of 7.0. Many women raised the smell of a product as the reason why they disliked or could not eat several products, notably many of the savoury products. For example, one focus group participant said of the seasoned pillows: ‘The odour makes it so that I can't even perceive the taste.’ In one focus group, participants discussed a pregnant woman's sensitivity to smell: ‘The smells that pregnant women smell other adults do not smell.’

#### 3.1.3 Familiarity

Focus group discussions revealed a positive correlation between the resemblance of a supplement to a known product and the appreciation the women had for that supplement. Some participants mentioned associations between products and specific local foods (such as couscous, porridge and others) or familiar ingredients or flavours (such as milk and peanut). In all these cases, the resemblance of products to favoured familiar foods was perceived as a positive influence on their opinion of a study product.

### 3.2 Use during pregnancy

Women expressed an intention to use all of the tested products during pregnancy, provided they were able to tolerate them. Some women said that they simply could not eat one or more of the products. For example, although the savoury version of the lipid‐based paste was the highest‐ranking product in the savoury product testing, some women reacted negatively to its smell, taste or texture. As one focus group participant put it, ‘It's good, but it's the salty taste that makes it so that I can't eat it.’ Another said, ‘When you eat it, it stays in your throat, it doesn't go down.’

Participants repeatedly referred to the products' health benefits as a driver of their intention to use them during pregnancy and intended to eat the products even if they disliked them. One woman stated regarding the chicken soup, ‘If I know … that it can have benefits for my health I'm going to do everything [I can] to drink it.’ Another participant said she disliked the savoury bar, but that she would still eat it: ‘I'll manage because it's a medicine.’ Although many women expressed dislike for the saltiness and odour of the savoury biscuit, they generally agreed that they would eat it daily for the benefit of their baby. As one woman concluded: ‘A medicine can't always have a good taste.’

Often women did not raise hedonic characteristics such as the taste or smell of the product as a factor influencing use or mentioned them only secondarily. In one focus group discussion, for example, only one of all the women who liked the product mentioned taste as a driver of her willingness to use it:

Participant 6: Because it's a medicine, I will eat it during my pregnancy.

Participant 4: I will eat it during my pregnancy because it's like a vitamin.

Participant 7: I will eat it during my pregnancy because it will take care of my baby.

Participant 1: For me, it's because it has a good taste.

Participant 2: What will make me eat it during my pregnancy is that it will take care of my baby and make it strong.

Participant 3: I will eat it during my pregnancy because it will make the baby in my belly grow.

With regard to the top two products, women consistently reported that they would eat both the lipid‐based paste and the vanilla biscuit throughout pregnancy, although their reasons differed: For some women, daily consumption in pregnancy was directly linked to taste, whereas others emphasised that the products were perceived to be a medicine with health benefits. Participants expressed a willingness to use both products daily during pregnancy even if they had to pay for it. The mean Likert score for willingness to use the vanilla biscuit daily if provided for free was 6.4 (SD = 0.8); for willingness to use daily if they had to pay for it, the mean score was 5.8 (SD = 1.3). Both of these were the highest scores received by any sweet product (if they had to pay, the vanilla biscuit was tied with the sweet lipid‐based paste).

### 3.3 Ease of use

Participants were asked to evaluate on the Likert scale the extent to which they agreed or disagreed that the products were easy to eat. The vanilla biscuit's score was higher than that of any other product at 6.5 (SD = 0.7). When discussed during focus groups, ease of consumption was found to relate specifically to the association of the vanilla biscuit with familiar products and to the health benefits perceived by the participants. For example, where all women agreed that the vanilla biscuit would be easy to consume, one participant explained, ‘It would be easy to eat because it resembles a biscuit and there are vitamins in it. It's good to eat.’ Participants also uniformly viewed the lipid‐based paste as easy to use and to carry, including for consumption away from home.

Participants also associated a product's health benefits with ease of preparation and ease of use at home and elsewhere. When asked about why the product was easy to use or prepare, a recurring response was that it was easy because of its taste or its health properties. For the lipid‐based paste, which requires no preparation and was well liked by participants, many women referred to the product's health benefits rather than ease of preparation or likeability as the main driver influencing their consumption. However, even products that required preparation (such as the vanilla drink and chicken soup) were characterised as easy to prepare and use at home and outside the home.

### 3.4 Sharing practices

The quantitative data demonstrated little variation in Likert scores regarding likelihood of sharing across the sweet products, with all scores ranging between 3.4 and 3.6 (where 1 indicates strong disagreement that they were likely to share and 7 indicates strong agreement). For the savoury products, likelihood of sharing ranged from 3.2 (for the bar) to 3.7 (for the biscuit and seasoned pillows).

Focus group questions on sharing focused on both the perceived expectation to share and the likelihood of sharing. Despite widespread reference to household members' expectations that the pregnant woman would share her food, a majority of women reported that they would not share the supplements. Reasons cited for why included ‘because it's reserved for pregnant women’, because ‘it's not a normal food’ and ‘because it has vitamins’ for pregnant women.

A minority of women said that they would share the product with others, particularly with other pregnant women or with children. There was also some indication in the qualitative data that participants might be more likely to share products they disliked; as one woman concluded, ‘Because I don't like it, if nevertheless someone wanted it, I would give it to them.’

A number of women anticipated having to hide the lipid‐based paste from children in order to avoid sharing. A participant described the intrahousehold sharing dynamic that she would face, explaining, ‘If it's me, you can't hide even if you're at home. There are people who if they see you eat, they will of course want it. There will be some who understand, they see the ‘burden’ that you carry [i.e. that you are pregnant], and at that time they'll think of that. But there are some who won't think, if they see you eat they'll think that it's for pleasure and they'll ask you. For me, that is the situation.’ There was consensus among participants that they would not be expected to reduce their share of the household's food as a result of having the supplements. The following statement was representative: ‘It's not going to make me lose my share of family food because we know that it's because of the pregnancy that we were given [the vanilla biscuit].’

## 4 DISCUSSION

The mixed methods approach used in this study revealed a number of factors significant to product acceptability and use. The quantitative data provided insight into factors affecting product preference such as hedonic characteristics and factors relating to future use. The qualitative data provided valuable contextual data about the reasons for preferences and the factors affecting future use. Key themes were perceived sweetness, odour and resemblance to familiar products as factors influencing product preference, perceptions of health benefits as a driver of use and sharing as a concern for future monitoring and sensitisation.

### 4.1 Factors influencing product acceptability

Research has shown that humans are attracted to sweet tastes, although this varies significantly among individuals as a result of factors such as age, race/ethnicity, nutritional deficiencies, and more (Drewnowski, Mennella, Johnson & Bellisle, 2012). Preferred tastes may also vary during pregnancy and may change depending on stage of pregnancy; an increased preference for savoury foods has been observed, for example, during the second and third trimesters of pregnancy (Weenen et al., 2019). Women in this study (mean gestational age, 5.3 months [SD = 1.9] had a strong preference for those products they perceived as sweet (compared with savoury/spicy versions of the tested products).

Odour also proved to be a significant factor in the present study. Physiologically, smell is closely linked to taste; increased olfaction during pregnancy has been documented, although the evidence is mostly anecdotal and conflicting (Cameron, 2014). In this study, sensitivity to smell appeared to be an important factor leading women to dislike or resist several products. Developers of nutritional supplements should thus be aware that it is important to test the hedonic properties, including odour, of any products in order to ensure acceptance of future users.

Finally, the familiarity of supplemental food flavours has been observed as a facilitator to product use (Ickes et al., 2012). As noted, several of the products developed for this study used locally familiar flavourings or ingredients. These products (mango flavoured and tomato–onion flavoured) were not among the most preferred by participants. However, other familiar flavours were singled out as influencing women's preference for certain products, such as the milk and peanut flavours identified in the top products. This points to the importance of hedonic testing and qualitative assessments of products and/or flavours that may be developed for use in the future.

### 4.2 Health benefits and product use and preferences

Previous studies have noted that perceived health benefits may influence acceptability and adherence in the context of maternal nutritional supplements (Klevor et al., 2016; Young, Blanco, Hernandez‐Cordero, Pelto, & Neufeld, 2010). Our analysis similarly revealed that participants valued the products' benefits for themselves and their unborn children. Women viewed the products as medicine more than food, and in focus groups, women indicated that this view was a significant factor in predicting product use.

### 4.3 Perceptions on sharing

Sharing has been widely recognised as a potential impediment to adherence in the context of nutritional supplements for children and adults (Ashorn et al., 2015; Cantrell et al., 2008; Flax et al., 2010; Ickes et al., 2012), and cultural expectations around food sharing can impact supplement use (Ickes et al., 2012). Findings from other studies have indicated that the estimated energy intake from supplements was at times lower than anticipated when sharing with other family members appeared to be the norm (Janmohamed et al., 2016). Sharing dynamics were therefore considered crucial for understanding the pregnant woman's perception of use of a product, particularly when likelihood to share would affect daily consumption. The results here indicate widespread expectations that a pregnant woman will share her food, especially with children, and a degree of pressure on her to do so. Although most women reported that they would not share the supplements, a minority of women said that they might do so. Sharing behaviour should thus be closely monitored, and additional data on sharing and expectations of sharing should be collected during the next phase of the study. Package design might also clearly state that the product is exclusively for pregnant women, in order to discourage sharing.

### 4.4 Study strengths and limitations

The present study provided valuable information for the remaining aspects of the overall study as well as for future product development. Study strengths included the consistency in findings across qualitative and quantitative data and the rich contextual information obtained to further explain quantitative results.

As is an inherent risk in rapid data collection, it is possible that participants expressed answers they perceived to be appropriate or socially desirable. Participants were encouraged to speak openly and honestly, and the frank and sincere dialogue elicited from participant discussions suggested that such bias was minimised. Findings were also triangulated across participant groups and with quantitative data to test the validity of answers.

Because of the volume of products, interactions with participants were sometimes quite long; despite the efforts of the facilitators, levels of participation engagement declined as the discussion continued. Where possible, focus group discussions were held in the morning to overcome issues of tiredness and fatigue.

It is important to acknowledge that the product preferences, and the women's intentions regarding future use and sharing, were expressed in the context of a single‐meal rapid assessment of the 12 products. Further evaluation during the next study (the second part of phase 1), where the two most preferred products will be used at home for 10 weeks, will provide rich information regarding the longer‐term acceptability of these products, as well as patterns of consumption and sharing within the family.

## 5 CONCLUSION

Women in this study had strong and relatively consistent opinions regarding product preference, clearly favouring products they perceived as sweet. The sweet lipid‐based paste and the vanilla biscuit were best liked overall. Women also favoured products that bore a resemblance to familiar, well‐liked foods. Participants' ability to tolerate products as well as product preferences appear to be driven in part by their perceptions of the odour of the products. Results also support the notion that hedonic properties, rather than convenience, will influence women's perceptions of how easy a product is to use at home and away. Nonetheless, it emerged from both the quantitative and the qualitative data that women intend to eat any product they can tolerate—regardless of how much they like it—because of the perceived benefits for their unborn child.

Information regarding women's intentions to share the selected products was mixed. Quantitative data suggested that women were somewhat unlikely to share any supplement they are provided in the future. Qualitative data suggest that women might be more likely to share with their children or other pregnant women or if they disliked the product. Women did express an understanding that the product was intended only for their use and an appreciation that it is to be considered a medicine, which also influenced expressed sharing intentions.

The results indicate that it may be advisable to focus future product development, and particularly the development of new flavour profiles, on favoured local tastes. In addition, a focus on minimising strong odours may be important for future prenatal product development. Understanding the challenges to targeting product use to pregnant women is essential for future use and distribution of nutritional supplements. This study demonstrates that potential sharing behaviours should be monitored and addressed in future parts of the MISAME III project.

## CONFLICTS OF INTEREST

LJ, BdK, KM, JB, KV, LCT, CL, NDC, MO, RG, PK and SI have no conflicts of interest to declare. SdP was involved with the development of one of the tested BEP products (the fermented drink) and identified the opportunity to include it among the products assessed in the study. Her involvement with the product's development did not influence analysis or interpretation of the results nor product selection; the fermented drink was not among those identified for potential inclusion in the clinical trial.

## CONTRIBUTIONS

LJ and KM wrote the paper. PK, CL, SI, SdP, NDC, JB, KM and LJ designed the study and the protocol. KM, LJ, NdC, BdK, KV, LCT and MO trained the field data collectors. MO and NdC directed the field data collection. RG oversaw field data collection. SI, KM, LJ, BdK and KV analysed the data. MO provided quality control for the data. All authors contributed to drafting the paper and have read and approved the final manuscript.

## Supporting information


**Table S1:** Target Nutrient Composition of Balanced Energy‐Protein Supplements
Supplementary Table 2: Added Sugars
