# Supplementary material for: Acceptability of 12 fortified balanced energy protein supplements ‐ Insights from Burkina Faso
Source: Matern Child Nutr. 2020 Aug 5;17(1):e13067. doi: 10.1111/mcn.13067 (PMC7729548; doi:10.1111/mcn.13067)
Supplement: Supplementary file 1 — Table S1: Target Nutrient Composition of Balanced Energy‐Protein Supplements Supplementary Table 2: Added Sugars [file MCN-17-e13067-s001.docx]

**SUPPLEMENTARY APPENDIX**

Supplementary Table 1: Target Nutrient Composition of Balanced Energy-Protein Supplements

| Total Energy | 250-500 kcal per serving |
| --- | --- |
| Fat Content | 10-60% of energy intake |
| Trans Fat Content | < 1% of energy intake |
| Protein Content | 16g (14-18g), digestible amino acid score (DIAAS) of ≥0.9 |
| Carbohydrates | No specific recommendations; depends on fat content of product type |
| Micronutrients | To include vitamins A, D, E, K, B1, B2, B3, B6, B9, B12 and C; iron, zinc, iodine, calcium, phosphorus, copper, selenium; optional: pantothenic acid, manganese, potassium, biotin and choline. Exact composition to be determined in line with specified ranges.  Minimum ranges: Estimated Average Requirements†  Maximum ranges: Recommended Daily Allowances† |

(Source: Bill & Melinda Gates Foundation, 2016, pp. 5-10)

† Certain exceptions apply; see source for detailed description of ranges.

Supplementary Table 2: Added Sugars

| **Product Name** | **Added Sugars/100g** |
| --- | --- |
| Sweet lipid-based spread | 18.0g |
| Mango bar | 21.6g |
| Vanilla Filled sticks | 20.0g |
| Vanilla biscuits | 19.9g |
| Vanilla drink | 13.7g |
| Unseasoned pillows† | 3.9g |
| Fermented drink† | 17.0g |
| Tomato and onion lipid-based spread | 2.0g |
| Tomato and onion bar | 13.0g |
| Tomato and onion biscuits | 0g |
| Chicken soup | 0g |
| Seasoned pillows | 5.1g |
